# The Burden and Spectrum of Vitreo-Retinal Diseases Among Ophthalmic Outpatients in a Resource-Deficient Tertiary Eye Care Setting in South-Eastern Nigeria

**DOI:** 10.4103/0974-9233.65491

**Published:** 2010

**Authors:** Boniface Ikenna Eze, Judith N. Uche, Jude O. Shiweobi

**Affiliations:** Department of Ophthalmology, University of Nigeria Teaching Hospital (UNTH), PMB 01139, Ituku-Ozalla, Enugu, Nigeria

**Keywords:** Nigeria, Ambulatory Care, Disease Rate and Pattern, Vitreo-Retinal Disease

## Abstract

**Purpose:**

This study was designed to determine the rate and pattern of vitreo-retinal diseases at a tertiary eye care center in South-eastern Nigeria.

**Materials and Methods:**

The outpatient register at the Eye Clinic of the University of Nigeria Teaching Hospital, Enugu, was retrospectively examined to identify all new patients registered between January 2004 and December 2008. A chart review of subjects with vitreo-retinal disease was conducted to record relevant demographic and clinical data including the needs for vitreo-retinal care. Descriptive and analytical statistics were performed. A *P*-value <0.001 (one degree of freedom) was considered statistically significant.

**Results:**

Of the 8,239 new patients reported during the period, 326 subjects (males- 59.3%; females- 40.7%; sex ratio = 1.1:1) aged 49.3 ± 16.8 years (range 3-82 years) had vitreo-retinal disease. The rate of vitreo-retinal disease was 3.9%. The rate was higher in subjects above 40 years old (*P* < 0.001), but did not differ between sexes (*P* = 0.469). Diabetic retinopathy (24.9%), hypertensive retinopathy (13.3%), and age-related macular degeneration (10.7%) were the leading vitreo-retinal diseases. Blindness from vitreo-retinal disease was bilateral in 6.1% of subjects and unilateral in 17.5% of subjects. The common co-morbidities were ocular conditions such as refractive error (19.8%), cataract (14.2%), and glaucoma (10.4%); and systemic conditions such as diabetes mellitus (14.6%) and hypertension (13.2%).

**Conclusions:**

The rate of vitreo-retinal diseases among new ophthalmic outpatients at UNTH, Enugu, is 3.9%. Retinal vascular disorders and age-related maculopathy are the leading retinal diseases. At UNTH, resource needs for vitreo-retinal care are urgent including retinal photography/angiography, laser photocoagulation, intra-vitreal pharmacotherapy, and vitreo-retinal surgery.

## INTRODUCTION

Pathologies of the posterior segment of the eye are common clinical conditions frequently encountered during routine ophthalmic workup. The management of these disorders presents peculiar diagnostic and therapeutic challenges, especially in the resource-deficient third world settings, due to scarcity of requisite human and material resources.

Recently, there has been a significant increase in the burden of vitreo-retinal disorders globally.^1^ In Nigeria, vitreo-retinal disorders constitute a significant cause of ocular morbidity and vision loss with reported hospital prevalence rates ranging from 4.5% to 13.0%.^2^–^4^ Elsewhere in Ethiopia,^5^ a 12.5% hospital prevalence of vitreo-retinal disorders was reported whereas a population-based survey in Iran^6^ documented a prevalence of 8.56%.

Previous reports from hospital-based studies,^7^,^8^ and general population surveys,^9^ of causes of low vision have implicated vitreo-retinal diseases as a major public eye health burden. To confront this emerging public health challenge, there is the obvious need to provide affordable and accessible diagnostic and therapeutic facilities, and appropriately trained ophthalmic personnels.^3^,^4^

To optimize the allocation of scarce eye care resources, there is a need for research data on the frequency and distribution of retinal diseases, related vision loss, and resource needs for adequate management.

In this study of vitreo-retinal disease in new ophthalmic outpatients at a tertiary care facility in Enugu, South-eastern Nigeria, we determined the burden, spectrum, and associated blindness and visual impairment, and the resource requirements for appropriate management and treatment.

## MATERIALS AND METHODS

Established in 1971, and located in the South-east geo-political zone of Nigeria, the University of Nigeria Teaching Hospital (UNTH), Enugu, is one of the six first generation teaching hospitals in Nigeria. Along with five other tertiary eye care centers in the South-east geo-political zone, UNTH provides curative and rehabilitative tertiary eye care services to the people living in the five component states of the zone and beyond.

The UNTH’s Ophthalmology Department has four outpatient clinics, an inpatient bed capacity of 27, and 1 operating room. Currently, there are 12 consultant ophthalmologists, 17 trainee ophthalmologists in various stages of their training programs, 5 optometrists, and 21 staff nurses. The consultant and trainee ophthalmologists are grouped into five clinical units which rotate daily in the outpatient clinic and operating room. Few consultant ophthalmologists have undergone formal short-term fellowship training in their subspecialties of interest; only one ophthalmologist, the author, has undergone a short-term fellowship in medical retina. The available equipment for the management of vitreo-retinal diseases includes two indirect ophthalmoscopes, one diode laser unit, and one Stratus OCT-TM (Optical coherence tomography, Zeiss-Meditec Inc, Jena, Germany) machine. There are no facilities for retinal photography/flourescein angiography, intra-vitreal pharmacotherapy, retinal cryopexy/scleral buckle, and posterior vitrectomy. Consequently, at the UNTH’s Ophthalmology Department, sub-specialty practice is still in its early stage of development.

The total number of new outpatients seen at the UNTH’s Eye clinic between January 2004 and December 2008 was obtained from the ophthalmic outpatient attendance register. The obtained list was examined to identify all new patients with diagnosis of vitreo-retinal disease; their clinical charts were recalled, and data on age, sex, clinical diagnosis, laterality of vitreo-retinal disease, ocular co-morbidities, and presenting and corrected distant visual acuities were noted. The human and material resource needs for adequate medical, para-surgical, i.e., laser and surgical management of the vitreo-retinal conditions was determined from the clinical diagnoses. Data were entered and analyzed with Graphpad Prism software, version 3.4 (GraphPad Software Inc. La Jolla, CA, USA). Simple descriptive statistics was used to generate frequencies, percentages, and proportions. Tests for significant inter-group differences were performed using the Chi-square test with a *P* < 0.001 (df =1) considered statistically significant.

The study was conducted in full compliance with the 1964 Helsinki declaration on research involving human subjects. Prior to commencement of the study, ethical clearance was obtained from the Ethics Committee (Institutional Review Board) of UNTH, Enugu.

## RESULTS

During the period January 2004 and December 2008, 8,239 new eye patients comprising 4,886(59.3%) males and 3,353 (40.7%) females were registered at the Ophthalmic Outpatient Department of the UNTH, Enugu. Of these, 326 (3.9%) new patients were diagnosed with vitreo-retinal disease. The cohort comprised 171 (52.5%) males and 155 (47.5%) females (male-to-female ratio = 1.1:1)—aged 49.3 ± 16.8 years (range 3–82 years. The cohort demographics are shown in Table 1. The rate of vitreo-retinal disease at the Ophthalmic Outpatient Department of UNTH was 3.9% (326/8,329). Vitreo-retinal disease was more common among subjects aged 40 years and older (83 vs. 238; Chi-square = 51.22, *P* < 0.001). However, there was no significant difference in prevalence between sexes (171 vs. 155; Chi-square =0.5237, *P* = 0.469).

**Table 1:** Cohort demographics of subjects with vitreo-retinal disease

Age group	Sex		Total(%)
	M	F	
0−10	6	3	9 (2.8)
11−20	13	6	19 (5.8)
21−30	18	10	28 (8.6)
31−40	16	16	32 (9.8)
41−50	31	37	68 (20.9)
51−60	36	46	82 (25.2)
61−70	38	27	65 (19.9)
71−80	11	10	21 (6.4)
81−90	2	0	2 (0.6)
Total (%)	171 (52.5)	155 (47.5)	326 (100.0)

M, male; F, female

Acquired retinopathies consisting of diabetic retinopathy (116 subjects, 24.9%), hypertensive retinopathy (62 subjects, 13.3%), and age-related macular degeneration (50 subjects, 10.7%) were the leading vitreo-retinal conditions. Vitreo-retinal disease was bilateral in 105 (32.2%) subjects and unilateral in 221 (67.8%) subjects.

Some subjects had different diseases in one or both eyes. Table 2 shows the distribution of ocular diagnosis.

**Table 2:** Ocular diagnosis of subjects with vitreo-retinal disease

Diagnosis	Number of eyes	Percentage
Diabetic retinopathy	116	24.9
Hypertensive retinopathy	62	13.3
Age-related macular	50	10.7
degeneration		
Retinochoroiditis	41	8.8
Retinitis pigmentosa	32	6.9
Retinal detachment	27	5.8
Retinoblastoma	13	3.1
Macular hole	14	3.0
HIV retinopathy	10	2.1
Retinal vascular occlusion	8	1.7
Posterior vitreous detachment	6	1.2
Sickle cell retinopathy	4	0.8
Solar retinopathy	4	0.8
Angiod streak	2	0.4
Unclassified retinopathy	49	10.5
Unclassified maculopathy	28	6.0
Total	466	100.0

The leading ocular co-morbidities were refractive error in 42 (19.8%) subjects, cataract in 30 (14.2%) and glaucoma in 22 (10.4%) subjects. Systemic co-morbidities included diabetes mellitus in 31 (14.6%) subjects and hypertension in 28 (13.2%) subjects [Table 3]. Some subjects had multiple ocular or systemic co-morbidities.

**Table 3:** Ocular and systemic co-morbidities

Disease	Number of subjects		Total(%) (*n* = 212)
	M	F	
Ocular			
Refractive error	18	24	42 (19.8)
Cataract	19	11	30 (14.2)
Glaucoma	13	9	22 (10.4)
Optic atrophy	2	1	3 (1.4)
Conjunctivitis	17	12	29 (13.7)
Pterygium	9	8	17 (8.0)
Systemic			
Diabetes mellitus	13	18	31 (14.6)
Hypertension	12	16	28 (13.2)
Peptic ulcer disease	5	2	7 (3.3)
Bronchial asthma	2	1	3 (1.4)
Total	110	102	212 (100.0)

Blindness from vitreo- retinal disease was bilateral in 20 (6.1%) subjects and unilateral in 57(17.5%) subjects.

Visual impairment was bilateral in 36(11.0%) subjects and unilateral in 68(20.9%) subjects.

Nineteen (5.8%) subjects were blind from ocular co-morbidity. Of these, blindness was bilateral in five subjects and unilateral in nine subjects.

Co-morbid eye disease caused bilateral visual impairment in 11 subjects and unilateral visual impairment in 6 subjects.

An assessment of diagnostic and therapeutic requirements showed that 69.7% of subjects needed fundus photography with or without fluorescein angiography, 20.1% of subjects required focal/grid/ pan-retinal green laser photocoagulation, 19.2% of subjects required intra-vitreal pharmacotherapy, 15.8% of subjects required posterior vitrectomy, 4.6% of subjects required cryopexy/scleral buckle, and 3.1% of subjects required radiotherapy.

## DISCUSSION

Vitreo-retinal disease was more common in males and in patients aged 40 years or older; however, the prevalence differed significantly by age, but not by sex. These findings are consistent with previous reports from similar studies elsewhere in Nigeria^3^,^4^ and Ethiopia^5^ but differ from the gender distribution documented in a population-based study in Iran.^6^ While the established pro-male gender bias in access to and uptake of eye care services^10^ may explain the observed male dominance, the age distribution could be attributed to the preponderance of age-related retinal diseases in this study. The similarity of the settings of the present survey with the Nigerian and Ethiopian studies, and its contrast with the Iranian survey might further account for these observations. These findings suggest that, to ensure equity in access to eye care, health care planners, administrators, and health care providers should identify and overcome gender- and age-related^11^ barrier to eye care services.

Compared with previous hospital based studies^2^–^5^, the 3.9% hospital prevalence of retinal diseases in a tertiary care center in this study is similar to 5.3%^2^ and 8.1%^3^ rates reported in Nigeria but lower than 13.0%^4^ reported from another survey in Nigeria and 12.5%^5^ in Ethiopia. The paucity of equipment and skills for the diagnosis of retinal diseases at the study center probably lead to under-diagnosis of these conditions thus accounting for the low prevalence rate in this study. Additionally, differences in methodologies between surveys might further explain the variations in rates of retinal diseases. This highlights the dire need for provision of requisite materials and manpower, at the study center, for accurate diagnosis of retinal diseases. Incorporating a mydriatic retinal examination, unless contra-indicated, into the routine examination protocol for ophthalmic outpatients will likely increase the detection of vitreo-retinal diseases.

Retinal vascular, age-related, and inflammatory retinopathies were the most common clinical conditions encountered in this study. This disease distribution is consistent with the patterns previously reported in Nigeria^3^,^4^ but contrasts the findings of Abiose^2^ in Lagos, and Teshome *et al*.^5^ in Ethiopia. While Abiose reported hereditary retinopathies, sickle cell retinopathy, and retinitis pigmentosa as the leading retinal pathologies, retinal detachment was the second most common retinal disease after retinal vascular disorders in the series reported by Teshome *et al*.^5^ The observed disparities could be attributed to time interval between studies and differences in genetic, racial, and geographic determinants of retinal disease in the study populations. These findings should enable decisions on resource allocation for optimal vitreo-retinal care in the catchment area of the study center.

Consistent with previous reports in Nigeria^3^ and Iran,^6^ in this study, cataract and glaucoma were common ocular co-morbidities whereas diabetes mellitus and systemic hypertension were the leading systemic co-morbidities. However, the Nigerian^3^ and Iranian^6^ studies failed to report on refractive error, the leading ocular co-morbidity in this study. The association of vitreo-retinal diseases with cataract and glaucoma, documented in this survey and elsewhere,^3^,^6^ could be explained by their common age-related etiologies. The established higher prevalence of retinal disease in patients with diabetes mellitus, systemic hypertension, and worse still in hypertensive diabetics probably explains the strong association of retinal disease with these conditions.^6^ These findings imply that planning for effective vitreo-retinal service delivery should incorporate strategies for the management of these ocular and systemic co-morbidities. The 6.1% prevalence of bilateral and 17.5% of unilateral blindness documented in this study are lower than the figures reported by Nwosu^3^ (14.0%, 40.0%) and Teshome *et al*.^5^ (11.0%, 20.9%). However, the prevalence of bilateral and unilateral visual impairment across the three studies are similar: present study – 11.0%, 20.9%, Nwosu’s – 16%, 16%, and Teshome *et al*. – 14%, 20.9%), respectively. The patients in this survey probably presented earlier, before the blinding stage diseases, possibly due to their high literacy and awareness level^2^ thus accounting for the comparatively low prevalence of blindness. This reinforces the suggestion made elsewhere^12^ for public education of patients with diabetes mellitus, the leading cause of vitreo-retinal disease in this study. Diabetic patients must be informed of the consequences of diabetic eye disease and the need for regular eye examination.

The main resources required for vitreo-retinal care in this survey were retinal photography/angiography, laser photocoagulation, and intra-vitreal pharmacotherapy. These results are similar to those identified by Onakpoya *et al*.,^4^ and Nwosu^3^ but differ from the overriding need for posterior vitrectomy suggested by Teshome *et al*.^5^ While Teshome and associates, in their survey, had retinal detachment as the prototype cause of blindness, they failed to mention how many of the detachments occurred on a background of medical retinal disease, especially diabetic retinopathy. Additionally, as previously stated, genetic, racial, and geographic factors could be responsible for the differences.

The retrospective nature of the study, its hospital-based setting, and deficient facilities for vitreo-retinal diagnosis, at the study center, were the limitations of this study. Future prospective population-based studies is needed to determine the true prevalence of vitreo-retinal disease in this population.

The prevalence of vitreo-retinal diseases among new ophthalmic outpatients at UNTH, Enugu, is 3.9%. Retinal vascular disease, age-related macular degeneration, and inflammatory retinopathies are the leading retinal diseases. The prevalence of blindness, but not visual impairment, from retinal disease is comparatively low. The need for resources such as fundus photography/fluorescein angiography, laser photocoagulation, and intra-vitreal pharmacotherapy are urgent.
